# Pancreatic GIST in a Patient with Limited Stage Small Cell Lung Cancer: A Case Report and Review of Published Cases

**DOI:** 10.1155/2016/9604982

**Published:** 2016-08-08

**Authors:** Minh Phan, Shari Jones, Justin Jenkins, Shubham Pant, Mohamad Khawandanah

**Affiliations:** ^1^Hematology-Oncology Section, Department of Medicine, The University of Oklahoma Health Sciences Center, Oklahoma City, OK 73104, USA; ^2^Department of Internal Medicine, The University of Oklahoma Health Sciences Center, Oklahoma City, OK 73104, USA; ^3^Department of Pathology, The University of Oklahoma Health Sciences Center, Oklahoma City, OK 73104, USA

## Abstract

Gastrointestinal stromal tumors (GISTs) are the most common mesenchymal tumors of the gastrointestinal tract and usually occur in the stomach and the small intestine. The pancreas is an extremely rare primary site for GISTs and there are 25 reported cases of pancreatic GIST with most being treated with surgical resection. We describe a 52-year-old African-American female who was diagnosed with limited stage small cell carcinoma in November 2009 and treated with concurrent cisplatin/etoposide chemotherapy and radiation. She subsequently achieved complete remission. Two years later she was diagnosed with localized pancreatic GIST by endoscopic ultrasonography guided fine needle aspiration. We treated her with a tyrosine kinase inhibitor (TKI) imatinib 400 mg oral dose daily as she declined surgery. Her disease is stable based on computed tomography imaging scans 40 months after diagnosis without any metastasis. To the best of our knowledge, our case is the second case of localized pancreatic GIST treated with TKI monotherapy.

## 1. Introduction

Gastrointestinal stromal tumors (GISTs) are the most common mesenchymal tumors of the gastrointestinal tract and usually occur in the stomach and the small intestine. The pancreas is an extremely rare primary site for GISTs. The annual incidence of GIST in the United States is 5000–6000/year and they are more common in the males, blacks, and the elderly [1, 2]. Workup of these lesions includes morphologic study and immunohistochemical and molecular diagnostic analysis. Historically, these neoplasms had been included under a number of diagnostic categories including leiomyoma, leiomyosarcoma, schwannoma, and leiomyoblastoma. Surgery was the only available treatment and this changed in 2001 after discovery of mutational activation of the KIT or PDGFRA genes [3] and the use of targeted therapies.

## 2. Methods

Abstracts, case reports, and case series of pancreatic GIST in the English literature were identified with no date limits until November 2015, by searching the keywords “pancreatic gastrointestinal tumors”, “pancreatic GIST”, and “extra gastrointestinal stromal tumors” in the National Library of Medicine, PubMed, OVID, and EMBASE search engines. Bibliographies of publications were also reviewed for additional relevant studies.

## 3. Case Presentation

**Figure 1:**
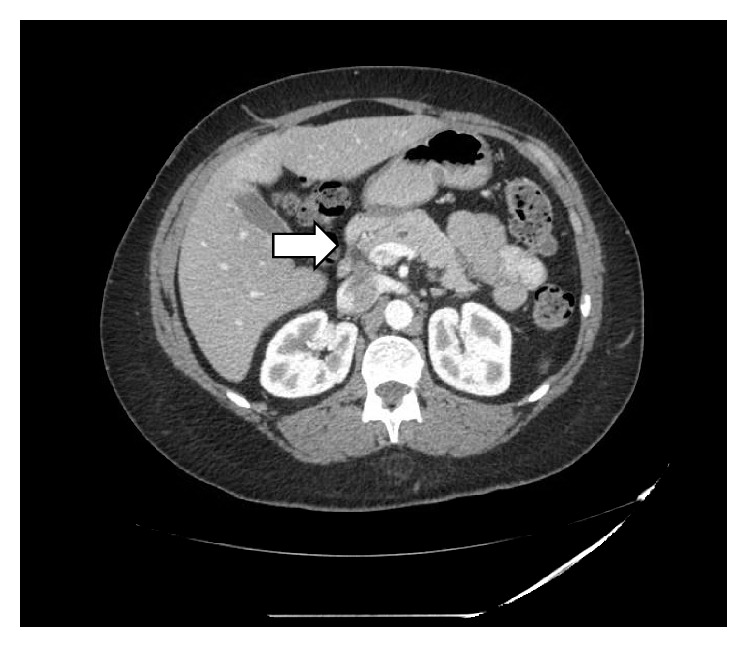
CT scan of the abdomen demonstrate a mass, arising from the uncinate process of the pancreas.

A 52-year-old African-American female was diagnosed with limited stage small cell carcinoma in November 2009 and treated with concurrent cisplatin/etoposide chemotherapy and radiation. She achieved complete remission and underwent prophylactic whole brain radiation in March 2010. Two years later she started to complain of vague abdominal pain and this was investigated with computed tomography (CT) scans which revealed a 3.5 cm enhancing lesion in the pancreas in addition to multiple uterine fibroids (Figure 1).

**Figure 2:**
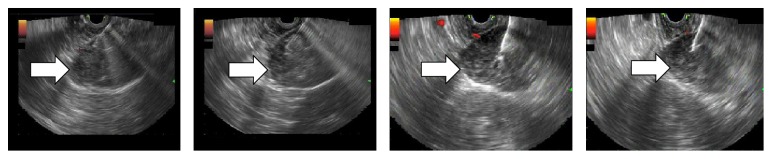
EUS showing hypoechoic mass in the pancreatic uncinate process during FNA procedure.

**Figure 3:**
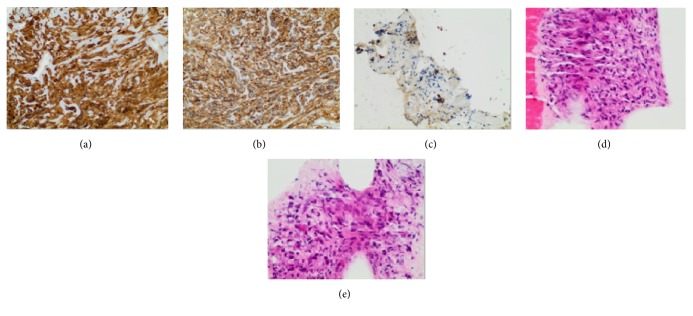
Pathology shows spindle cell lesion composed of intersecting fascicles and relatively bland spindle cells. (a) CD117, (b) DOG1, (c) spindled tumor cells which are negative for pancytokeratin, (d) atypical spindle cells on H&E stain, and (e) atypical spindle cells on H&E stain.

She underwent endoscopic ultrasonography guided fine needle aspiration (EUS-FNA) of the pancreatic lesion (Figure 2). Cytopathology revealed atypical cells with spindle cell features. Another EUS-FNA along with core biopsy sampling was performed, yielding the pathological diagnosis of gastrointestinal stromal tumor. Immunohistochemical staining of stromal cells was positive for CD117 (c-kit) and DOG-1 and negative for smooth muscle actin, S-100 protein, and ALK-1 (Figure 3 and Table 1).

We proceeded with medical therapy as patient declined surgical approach, and she was started on treatment with imatinib 400 mg PO daily. During treatment, she experienced imatinib side effects including nausea, vomiting, and leg cramps; we controlled these with promethazine and carisoprodol. Her disease is stable based on CT scans 40 months after diagnosis without any evidence of metastatic disease.

## 4. Discussion

GISTs are group of tumors showing differentiation or derived from the interstitial cells of Cajal which works as the GI pacemaker cells and like GISTs, these cells express both KIT and CD34 [4, 5]. Eighty percent of GIST cases have a mutation in the KIT gene exon 8, 9, 11, 13, or 17 [6]. In around 7% of cases there are mutations in PDGFR exon 12, 14, 18 D842V, or 18 [7].

Rarely, wild-type adult GIST tumors are associated with activation of the succinate dehydrogenase (SDH) complex like cases of GIST associated with Carney triad or Neurofibromatosis 1 [8]. On the other hand, wild type is very common in pediatric GIST [9] in around 85% cases while only 10–15% of adult cases do not harbor any mutation in the KIT and PDGFR genes [10].

GISTs commonly involve the stomach (60%), jejunum and ileum (30%), duodenum (4-5%), rectum (4%), colon and appendix (1-2%), and esophagus (<1%) and rarely present as primary tumors outside the gastrointestinal lumen such as the omentum, mesentery, and urinary bladder [11–13] or as in our case the pancreas. Both extragastrointestinal GIST and GIST are thought to originate from the gut smooth muscle cells and interstitial cells of Cajal; the former is thought to contribute to the growth outside of the gastrointestinal tract [14]. Another theory is that extragastrointestinal GISTs are mural GISTs which result in extramural growth [14].

The incidence of GIST is around eleven per million population in an Icelandic study [15]. It is difficult to determine the incidence due to the rarity of extragastrointestinal GIST. The mean age at diagnosis was 63 [1] for GIST compared to 53 for pancreatic GIST from the reviewed case reports. Gender involvement was not different between the pancreatic and extragastrointestinal GIST. A formal statistical analysis was not performed with the available case report data.

GISTs display two morphologic variants represented by the spindle cell and epithelioid subtypes. The spindle cell type is the most frequent, while the histological patterns relate to site of primary origin [16]. The majority of GISTs are strongly positive when stained with antibodies directed against the KIT protein (CD117), and the combination of CD34 and CD117 positivity aids in confirmation of the diagnosis of GIST. There are two targets that have been found to be useful in the diagnosis of GISTs: both C (PKC)-O and DOG1 are expressed in KIT positive and KIT negative GIST [17, 18].

Mutational analysis can aid in determining prognosis or if GIST will be responsive to imatinib therapy, it can also predict which dose level is most appropriate [19, 20]. For example, exon 9 mutant tumors carry the worst prognosis but have superior objective response to tumors with mutations in exon 11, and those patients with documented exon 9 mutations benefit from an 800 mg dose of imatinib rather than the standard 400 mg PO daily dose [21]. Routine mutation analysis is not recommended by the National Comprehensive Cancer Network (NCCN) GIST Task Force due to insufficient data for risk stratification and relapse prognostication [22]. Because of this report, we did not perform the mutation analysis on our patient. This is in contrast to the European Society for Medical Oncology guidelines which support administering an imatinib dose of 800 mg daily for exon 9 mutation [23]. The imatinib 400 mg regimen was chosen due to the Gastrointestinal Stromal Tumor Meta-Analysis Group data [24].

While imatinib can be used in the neoadjuvant or adjuvant setting, sunitinib—which is another tyrosine kinase inhibitor (TKI)—is frequently used as second-line therapy in refractory disease or in case of imatinib intolerance [25]. Sunitinib is administered at 50 mg starting dose in 6-week cycles with 4 weeks on and 2 weeks off treatment and can be also given as 37.5 mg PO daily which appears safe and effective [26]. Regorafenib is a TKI that targets multiple kinases including PDGFR, KIT, and vascular endothelial growth factor receptors; it can be used in advanced GIST after failure of both imatinib and sunitinib [27]. In the third-line setting, other TKIs such as sorafenib and nilotinib have significant clinical activity in imatinib and sunitinib resistant GIST and may represent an alternative for rechallenge treatment with imatinib, which is of limited benefit; nevertheless, it is superior to best supportive care in terms of overall survival [28]. Ganjoo et al. reported the use of pazopanib, another TKI, in a phase 2 clinical trial as a single agent with marginal activity in unselected heavily pretreated patients with advanced GIST [29].

To the best of our knowledge, our case is unique in terms of long survival with single nonsurgical modality and it is the second case of localized pancreatic GIST treated only with TKI. In the English literature there are 25 reported cases of pancreatic GIST (Table 2). Padhi et al. reported nineteen cases of pancreatic GIST gathered from 2000 to 2012 [30]. In 2015, Joseph et al. reported a case of a patient with pancreatic GIST that was started on imatinib but later developed metastatic disease and died 9 months later [31]. If the tumor can be resected, then treatment of choice would be surgery. The stabilization of the lesion in our patient with TKI therapy suggests that this is a reasonable therapeutic course in patients who are not surgical candidates. The lesion should be reevaluated for resection within three to four months [56]. Given the limited long term follow-up of patients with the pancreas as the site of origin, it is unclear whether pancreatic GISTs have a different natural history relative to luminal GISTs.

## Figures and Tables

**Table 1:** Immunohistochemical stains performed on our pancreatic GIST case.

Stain	Result
CD117 (c-KIT)	Strongly and diffusely positive in spindle cells
DOG-1	Strongly and diffusely positive in spindle cells
Smooth muscle actin (SMA)	Negative in spindle cells
S-100 protein	Negative in spindle cells
ALK-1	Negative in spindle cells

**Table 2:** List of published cases in English literature of pancreatic GIST.

Number of patients	Age	Gender	Primary treatment	Clinical presentation	Year of report	Author	Reference
1	54	Female	Surgery	Abdominal tumor	2004	Yamaura et al.	[32]
1	48	Female	Surgery	Asymptomatic abdominal mass	2004	Neto et al.	[33]
1	38	Female	Surgery	Not described	2005	Krska et al.	[34]
1	70	Female	Surgery	Asymptomatic abdominal mass	2005	Daum et al.	[35]
1	47	Male	n/a	Nausea/vomiting	2008	Yan et al.	[36]
1	55	Male	Surgery	Poor appetite, abdominal discomfort	2008	Yang et al.	[37]
1	63	Female	Surgery	Flank pain	2009	Harindhanavudhi et al.	[38]
1	58	Male	Surgery	Weight loss, dysuria	2009	Goh et al.	[39]
1	52	Female	Surgery	Epigastric pain	2009	Trabelsi et al.	[40]
1	42	Male	Surgery	Asymptomatic abdominal mass	2011	Meng et al.	[41]
1	42	Female	Surgery	Abdominal pain, loss of appetite, weight	2010	Padhi et al.	[42]
1	31	Male	Surgery	Abdominal pain, fatigue, weight loss	2010	Saif et al.	[43]
1	61	Male	Surgery	Fever, sweating, weight loss	2010	Crisan et al.	[44]
1	84	Male	Supportive	Abdominal distension, confusion, agitation	2010	Joshi and Rustagi	[45]
1	n/a	n/a	Surgery	n/a	2011	Barros et al.	[46]
1	74	Female	Surgery	Abdominal mass	2011	Čečka et al.	[47]
1	40	Male	Surgery	Athenia, abdominal pain, low grade fever, severe anemia, loss of appetite, weight loss	2011	Rao et al.	[48]
1	55	Male	Surgery	Postprandial abdominal discomfort	2012	Kim et al.	[49]
1	55	Female	Surgery	Abdominal pain	2012	Babu et al.	[50]
1	39	Male	Surgery	Weight loss, epigastric pain, constipation, anorexia	2013	Soufi et al.	[51]
1	30	Male	Surgery	Abdominal distension	2013	Serin et al.	[52]
1	74	Female	Surgery	Gastrointestinal bleeding	2014	Hansen et al.	[53]
1	56	Male	Surgery	Gastrointestinal hemorrhage, abdominal pain	2015	Aziret et al.	[54]
1	55	Male	Surgery	Asymptomatic	2015	Stanek et al.	[55]
1	60	Male	Medical	Abdominal pain	2015	Joseph et al.	[31]

## References

[1] Tran T., Davila J. A., El-Serag H. B. (2005). The epidemiology of malignant gastrointestinal stromal tumors: an analysis of 1,458 cases from 1992 to 2000. *American Journal of Gastroenterology*.

[2] Fletcher C. D. M., Berman J. J., Corless C. (2002). Diagnosis of gastrointestinal stromal tumors: a consensus approach. *Human Pathology*.

[3] DeMatteo R. P., Lewis J. J., Leung D., Mudan S. S., Woodruff J. M., Brennan M. F. (2000). Two hundred gastrointestinal stromal tumors: recurrence patterns and prognostic factors for survival. *Annals of Surgery*.

[4] Fletcher C. D. M., Berman J. J., Corless C. (2002). Diagnosis of gastrointestinal stromal tumors: a consensus approach. *International Journal of Surgical Pathology*.

[5] Hirota S., Isozaki K., Moriyama Y. (1998). Gain-of-function mutations of c-kit in human gastrointestinal stromal tumors. *Science*.

[6] Emile J. F., Theou N., Tabone S. (2004). Clinicopathologic, phenotypic, and genotypic characteristics of gastrointestinal mesenchymal tumors. *Clinical Gastroenterology and Hepatology*.

[7] Corless C. L., Schroeder A., Griffith D. (2005). PDGFRA mutations in gastrointestinal stromal tumors: frequency, spectrum and in vitro sensitivity to imatinib. *Journal of Clinical Oncology*.

[8] Janeway K. A., Kim S. Y., Lodish M. (2011). Defects in succinate dehydrogenase in gastrointestinal stromal tumors lacking KIT and PDGFRA mutations. *Proceedings of the National Academy of Sciences of the United States of America*.

[9] Prakash S., Sarran L., Socci N. (2005). Gastrointestinal stromal tumors in children and young adults: a clinicopathologic, molecular, and genomic study of 15 cases and review of the literature. *Journal of Pediatric Hematology/Oncology*.

[10] Nannini M., Biasco G., Astolfi A., Pantaleo M. A. (2013). An overview on molecular biology of KIT/PDGFRA wild type (WT) gastrointestinal stromal tumours (GIST). *Journal of Medical Genetics*.

[11] Miettinen M., Lasota J. (2006). Gastrointestinal stromal tumors: pathology and prognosis at different sites. *Seminars in Diagnostic Pathology*.

[12] Lasota J., Carlson J. A., Miettinen M. (2000). Spindle cell tumor of urinary bladder serosa with phenotypic and genotypic features of gastrointestinal stromal tumor. *Archives of Pathology and Laboratory Medicine*.

[13] Miettinen M., Monihan J. M., Sarlomo-Rikala M. (1999). Gastrointestinal stromal tumors/smooth muscle tumors (GISTs) primary in the omentum and mesentery: clinicopathologic and immunohistochemical study of 26 cases. *The American Journal of Surgical Pathology*.

[14] Beltrame V., Gruppo M., Pastorelli D., Pizzi S., Merigliano S., Sperti C. (2014). Extra-gastrointestinal stromal tumor of the pancreas: case report and review of the literature. *World Journal of Surgical Oncology*.

[15] Tryggvason G., Gíslason H. G., Magnússon M. K., Jónasson J. G. (2005). Gastrointestinal stromal tumors in Iceland, 1990–2003: the Icelandic GIST study, a population-based incidence and pathologic risk stratification study. *International Journal of Cancer*.

[16] Layfield L. J., Wallander M. L. (2012). Diagnosis of gastrointestinal stromal tumors from minute specimens: cytomorphology, immunohistochemistry, and molecular diagnostic findings. *Diagnostic Cytopathology*.

[17] Espinosa I., Lee C.-H., Kim M. K. (2008). A novel monoclonal antibody against DOG1 is a sensitive and specific marker for gastrointestinal stromal tumors. *The American Journal of Surgical Pathology*.

[18] Motegi A., Sakurai S., Nakayama H., Sano T., Oyama T., Nakajima T. (2005). PKC theta, a novel immunohistochemical marker for gastrointestinal stromal tumors (GIST), especially useful for identifying KIT-negative tumors. *Pathology International*.

[19] Kontogianni-Katsarou K., Dimitriadis E., Lariou C., Kairi-Vassilatou E., Pandis N., Kondi-Paphiti A. (2008). KIT exon 11 codon 557/558 deletion/insertion mutations define a subset of gastrointestinal stromal tumors with malignant potential. *World Journal of Gastroenterology*.

[20] Corless C. L., Heinrich M. C. (2008). Molecular pathobiology of gastrointestinal stromal sarcomas. *Annual Review of Pathology: Mechanisms of Disease*.

[21] Debiec-Rychter M., Sciot R., Le Cesne A. (2006). KIT mutations and dose selection for imatinib in patients with advanced gastrointestinal stromal tumours. *European Journal of Cancer*.

[22] Demetri G. D., von Mehren M., Antonescu C. R. (2010). NCCN Task Force report: update on the management of patients with gastrointestinal stromal tumors. *Journal of the National Comprehensive Cancer Network*.

[23] European Sarcoma Network Working Group (2014). Gastrointestinal stromal tumours: ESMO clinical practice guidelines for diagnosis, treatment and follow-up. *Annals of Oncology*.

[24] Van Glabbeke M. (2010). Comparison of two doses of imatinib for the treatment of unresectable or metastatic gastrointestinal stromal tumors: a meta-analysis of 1,640 patients. *Journal of Clinical Oncology*.

[25] Demetri G. D., van Oosterom A. T., Garrett C. R. (2006). Efficacy and safety of sunitinib in patients with advanced gastrointestinal stromal tumour after failure of imatinib: a randomised controlled trial. *The Lancet*.

[26] George S., Blay J. Y., Casali P. G. (2009). Clinical evaluation of continuous daily dosing of sunitinib malate in patients with advanced gastrointestinal stromal tumour after imatinib failure. *European Journal of Cancer*.

[27] George S., Wang Q., Heinrich M. C. (2012). Efficacy and safety of regorafenib in patients with metastatic and/or unresectable GI stromal tumor after failure of imatinib and sunitinib: a multicenter phase II trial. *Journal of Clinical Oncology*.

[28] Italiano A., Cioffi A., Coco P. (2012). Patterns of care, prognosis, and survival in patients with metastatic gastrointestinal stromal tumors (GIST) refractory to first-line imatinib and second-line sunitinib. *Annals of Surgical Oncology*.

[29] Ganjoo K. N., Villalobos V. M., Kamaya A. (2014). A multicenter phase II study of pazopanib in patients with advanced gastrointestinal stromal tumors (GIST) following failure of at least imatinib and sunitinib. *Annals of Oncology*.

[30] Padhi S., Sarangi R., Mallick S. (2013). Pancreatic extragastrointestinal stromal tumors, interstitial Cajal like cells, and telocytes. *Journal of the Pancreas*.

[31] Joseph P., Goyal R., Bansal P., Parmar R., Dutt S. (2015). Pancreatic extra-gastrointestinal stromal tumour with documentation of C-Kit mutation: a case report. *Journal of Clinical and Diagnostic Research*.

[32] Yamaura K., Kato K., Miyazawa M. (2004). Stromal tumor of the pancreas with expression of c-kit protein: report of a case. *Journal of Gastroenterology and Hepatology*.

[33] Neto M. R. M., Machuca T. N., Pinho R. V., Yuasa L. D., Bleggi-Torres L. F. (2004). Gastrointestinal stromal tumor: report of two unusual cases. *Virchows Archiv*.

[34] Krska Z., Peskova M., Povysil C. (2005). GIST of pancreas. *Prague Medical Report*.

[35] Daum O., Klecka J., Ferda J. (2005). Gastrointestinal stromal tumor of the pancreas: case report with documentation of KIT gene mutation. *Virchows Archiv*.

[36] Yan B. M., Pai R. K., Van Dam J. (2008). Diagnosis of pancreatic gastrointestinal stromal tumor by EUS guided FNA. *Journal of the Pancreas*.

[37] Yang F., Long J., Di Y. (2008). A giant cystic lesion in the epigastric region. *Gut*.

[38] Harindhanavudhi T., Tanawuttiwat T., Pyle J., Silva R. (2009). Extra-gastrointestinal stromal tumor presenting as hemorrhagic pancreatic cyst diagnosed by EUS-FNA. *Journal of the Pancreas*.

[39] Goh B. K. P., Kesavan S. M., Wong W.-K. (2009). An unusual cause of a pancreatic head tumor. *Gastroenterology*.

[40] Trabelsi A., Yacoub-Abid L. B., Mtimet A. (2009). Gastrointestinal stromal tumor of the pancreas: a case report and review of the literature. *North American Journal of Medical Sciences*.

[41] Meng L., Fang S.-H., Jin M. (2011). An unusual case of pancreatic and gastric neoplasms (2010: 12b). Malignant GISTs originating from the pancreas and stomach. *European Radiology*.

[42] Padhi S., Kongara R., Uppin S. G. (2010). Extragastrointestinal stromal tumor arising in the pancreas: a case report with a review of the literature. *Journal of the Pancreas*.

[43] Saif M. W., Hotchkiss S., Kaley K. (2010). Gastrointestinal stromal tumors of the pancreas. *Journal of the Pancreas*.

[44] Crisan A., Nicoara E., Cucui V., Cornea G., Laza R. (2010). Prolonged fever associated with gastrointestinal stromal tumor—case report. *Journal of Experimental Medical & Surgical Research*.

[45] Joshi J., Rustagi T. (2010). Pancreatic extra-gastrointestinal stromal tumor: an unusual presentation of a rare diagnosis. *Gastrointestinal Cancer Research*.

[46] Barros A., Linhares E., Valadão M. (2011). Extragastrointestinal stromal tumors (EGIST): a series of case reports. *Hepato-Gastroenterology*.

[47] Čečka F., Jon B., Ferko A., Šubrt Z., Nikolov D. H., Tyčová V. (2011). Long-term survival of a patient after resection of a gastrointestinal stromal tumor arising from the pancreas. *Hepatobiliary and Pancreatic Diseases International*.

[48] Rao R. N., Vij M., Singla N., Kumar A. (2011). Malignant pancreatic extra-gastrointestinal stromal tumor diagnosed by ultrasound guided fine needle aspiration cytology. A case report with a review of the literature. *Journal of the Pancreas*.

[49] Kim H.-H., Koh Y.-S., Park E.-K. (2012). Primary extragastrointestinal stromal tumor arising in the pancreas: report of a case. *Surgery Today*.

[50] Babu S. R., Kumari S., Zhang Y., Su A., Wang W., Tian B. (2012). Extra gastrointestinal stromal tumor arising in the pancreas: a case report and literature review. *Journal of Gastroenterology and Hepatology Research*.

[51] Soufi M., Bouziane M., Massrouri R., Chad B. (2013). Pancreatic GIST with pancreas divisum: a new entity. *International Journal of Surgery Case Reports*.

[52] Serin K. R., Keskin M., Güllüoğlu M., Emre A. (2013). Atypical localisation of a gastrointestinal stromal tumour: a case report of pancreas gastrointestinal stromal tumour. *Ulusal Cerrahi Dergisi*.

[53] Hansen C. A. P., José F. F., Caluz N. P. (2014). Gastrointestinal stromal tumor (GIST) mistaken for pancreatic pseudocyst—case report and literature review. *Clinical Case Reports*.

[54] Aziret M., Çetinkünar S., Aktaş E., İrkörücü O., Bali İ., Erdem H. (2015). Pancreatic gastrointestinal stromal tumor after upper gastrointestinal hemorrhage and performance of whipple procedure: a case report and literature review. *American Journal of Case Reports*.

[55] Stanek M., Pędziwiatr M., Matłok M., Budzyński A. (2015). Laparoscopic removal of gastrointestinal stromal tumors of uncinate process of pancreas. *Wideochirurgia I Inne Techniki Maloinwazyjne*.

[56] Bormann F., Wild W., Aksoy H., Dörr P., Schmeck S., Schwarzbach M. (2014). A pancreatic head tumor arising as a duodenal GIST: a case report and review of the literature. *Case Reports in Medicine*.

